# Prospects and Challenges of Phospholipid-Based Prodrugs

**DOI:** 10.3390/pharmaceutics10040210

**Published:** 2018-11-01

**Authors:** Milica Markovic, Shimon Ben-Shabat, Shahar Keinan, Aaron Aponick, Ellen M. Zimmermann, Arik Dahan

**Affiliations:** 1 Department of Clinical Pharmacology, School of Pharmacy, Faculty of Health Sciences, Ben-Gurion University of the Negev, Beer-Sheva 8410501, Israel; milica@post.bgu.ac.il (M.M.); sbs@bgu.ac.il (S.B.-S.); 2 Cloud Pharmaceuticals Inc., Durham, NC 27709, USA; skeinan@cloudpharmaceuticals.com; 3 Department of Chemistry, University of Florida, Gainesville, FL 32603, USA; aaron.aponick@gmail.com; 4 Department of Medicine, Division of Gastroenterology, University of Florida, Gainesville, FL 32610, USA; Ellen.Zimmermann@medicine.ufl.edu

**Keywords:** drug delivery, drug therapy, phospholipids, prodrugs, PLA_2_

## Abstract

Nowadays, the prodrug approach is used already at the early stages of drug development. Lipidic prodrug approach is a growing field for improving a number of drug properties/delivery/therapy aspects, and can offer solutions for various unmet needs. This approach includes drug moiety bound to the lipid carrier, which can be triglyceride, fatty acids, steroid, or phospholipid (PL). The focus of this article is PL-based prodrugs, which includes a PL carrier covalently bound to the active drug moiety. An overview of relevant physiological lipid processing pathways and absorption barriers is provided, followed by drug delivery/therapeutic application of PL-drug conjugates, as well as computational modeling techniques, and a modern bioinformatics tool that can aid in the optimization of PL conjugates. PL-based prodrugs have increased lipophilicity comparing to the parent drug, and can therefore significantly improve the pharmacokinetic profile and overall bioavailability of the parent drug, join the endogenous lipid processing pathways and therefore accomplish drug targeting, e.g., by lymphatic transport, drug release at specific target site(s), or passing the blood-brain barrier. Moreover, an exciting gateway for treating inflammatory diseases and cancer is presented, by utilizing the PL *sn*-2 position in the prodrug design, aiming for PLA_2_-mediated activation. Overall, a PL-based prodrug approach shows great potential in improving different drug delivery/therapy aspects, and is expected to grow.

## 1. Introduction

Prodrugs are bioreversible derivatives of active drug molecules used to overcome certain physiological barriers [1]. Prodrugs are used to optimize the features of the parent drug, such as physicochemical, ADME (absorption, distribution, metabolism, excretion) and pharmacological properties. The prodrug approach can facilitate formulation and administration, drug efficacy, improves site specificity, and/or decrease toxicity [2,3].

The prodrug design encompases two different approaches: Hydrophilic prodrugs (where the prodrug has lower lipophilicity than the parent drug) [4] and lipophilic prodrugs (where the prodrug is more lipophilic than the parent drug) [5]. Lipidic prodrugs consist of two main parts: The drug moiety covalently attached to the lipid carrier, where the lipid carrier can be a fatty acid (FA), glyceride, steroid, or phospholipid (PL) [6]. A major strength of lipidic prodrug approach is that a carefully designed lipidic prodrug can take advantage of physiological lipid metabolic pathways and efficiently deliver the prodrug across barriers that are difficult to overcome otherwise [7].

The focus of this work is the PL-based prodrugs approach; it is aimed to overview trends and uses, prospects and challenges, promising therapeutic applications, as well as modelling methods for improving their design. The traditional prodrug approach focuses on altering the physicochemical drug properties by covalently binding the drug to hydrophilic or lipophilic moiety thereby increasing drug solubility [4] or passive permeability [8]. In contrast, the modern prodrug approach focuses on enabling site specificity, by targeting a specific membrane transporter or an enzyme. This “targeted” prodrug approach accounts for molecular and cellular factors such as influx/efflux of membrane transporters or cellular protein expression and distribution, thereby providing the opportunity for drug targeting, improving oral bioavailability, or selective organ/tissue activation [8,9,10,11,12,13]. The PL-prodrug approach is often based on targeting phospholipase A_2_ (PLA_2_), an enzyme that hydrolyzes the *sn*-2 fatty acyl bond of PL, thereby liberating lysophospholipid (LPL) and a free FA. Secretory phospholipase A_2_ (sPLA_2_) is overexpressed in the tissues of various inflammatory and malignant diseases [14,15,16,17,18,19], and PL-based prodrugs susceptible to sPLA_2_ is one of the main foci of this work. PLA_2_ does not have specific fatty acid selectivity, hence an intelligent design of PL-drug conjugate can result in successful PLA_2_-mediated activation and consequent release of the parent drug from the conjugate complex at the site of overexpression. On the other hand, in some cases it may be desired to avoid PLA_2_-mediated prodrug activation, therefore the phosphate group of the PL is subjected to conjugation, offering different pharmacological profile, possibility of passing the blood-brain barrier (BBB), and bypassing multidrug resistance mechanisms in antiviral and cancer treatment, but at the same time bypassing GI activation by PLA_2_. The use of PLs in lipid-based delivery systems are outside of the scope on this paper.

The design and structure of different PL-based conjugates, and the relevant physiological PL-processing pathways, will be covered hereinafter. Next, we will focus on innovations and therapeutic applications that were shown as promising up to date, including structure-activity relationship and computational modeling. Overall, we aim to provide insights into the use of this approach that continuously grows and may aid in the development of better drug products.

## 2. Processing Pathways of Phospholipids and Phospholipid-Based Prodrugs

PLs within the intestinal lumen are originated from nutritional and biliary sources. PLs are not absorbed intact; prior to absorption, the stereospecific enzyme PLA_2_ hydrolyses the *sn*-2 positioned fatty acid of the PL thereby releasing the free FA and *sn*-1 LPL, which are then ready for absorption. Once they are absorbed into the enterocytes, LPL is re-acylated to PL by the action of the enzyme lysophosphatidylcholine acyltransferase, and due to the amphiphilic nature of the PL it is now involved with stabilization of the surface of lipoproteins, produced *in-situ* within the enterocyte [20,21]. Lipoproteins constitute of triglyceride and cholesterol ester hydrophobic core, and a more hydrophilic surface which constitutes primarily of PL, free cholesterol and apolipoproteins. The main classes of lipoproteins are chylomicrons and very low-density lipoproteins [21]. Lipoproteins are synthetized in the rough endoplasmic reticulum, packaged in the Golgi apparatus, and undergo exocytosis by fusion with the basolateral enterocyte membrane and release into the interstitial space. After reaching the lamina propria, it favors transport into the open capillaries of the mesenteric lymphatic vessel called lacteals, rather than blood capillaries [22].

Attributable to structure/configuration similarity to LPL, highly lipophilic prodrugs may be conveyed into the intestinal lymphatic system as well [23]. It is estimated that log *P* value above 5, and solubility in TG of >50 mg/g are prerequisites for intestinal lymphatic transport [24]. Similarly to LPs, the PL-drug complex, which is now part of the lipoprotein surface, can be absorbed from the lamina propria into the porous mesenteric lymphatic vessel capillary lacteal, rather than blood capillaries; the porous structure of the lacteal and the absence of basal membrane allows permeation of large colloids (200–800 mm), whereas blood capillaries have tight junctions and continuous basal membrane that limits the permeation of large colloids [25]. Lacteals and submucosal lymphatic vessels make up the lymphatic efferent trunks and consequently the thoracic duct, which enters the systemic blood circulation at the junction of left internal jugular and subclavian veins, and by doing so bypasses the portal blood [26]. This allows highly lipophilic drugs/prodrugs to bypass first-pass hepatic metabolism and makes them orally bioavailable, allowing the possibility of altering the drug delivery rate to the blood and controlled drug delivery. The study with dipalmitoylphosphatidylfluorouridine prodrug demonstrated that after oral administration, the prodrug is absorbed from the intestinal tract via the deacylation-reacylation cycle for the uptake of phospholipids, and is then selectively delivered through the lymphatic route [27]. In some cases prodrugs that include direct conjugation between PL and the drug moiety, can be absorbed into enterocytes intact and undergo lymphatic transport [28].

The structure and design of the PL prodrugs can highly influence their fate within the body. Careful design can direct the complex to the desired processing pathway, aiming to achieve different purposes, e.g., lymphatic transport [28], controlled release [29], local effect at the site of inflammation [30,31,32], and others.

## 3. Phospholipid-Based Prodrugs: Structures and Applications

**Figure 1:**
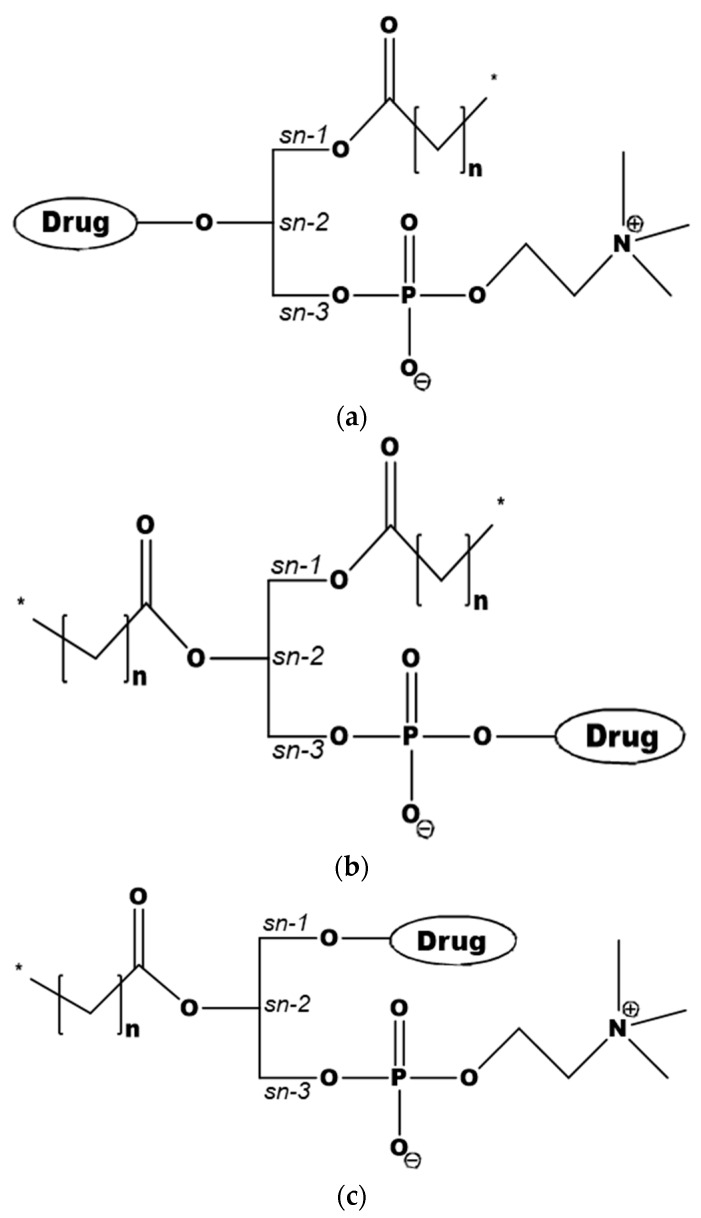
PL-drug conjugates, with the drug moiety attached to the *sn*-2 position of the glycerol backbone (**a**), to the phosphate group (**b**), or to the *sn*-1 position (**c**).

The design of PL-based prodrugs includes either the active drug moiety linked to the phosphate group, or the drug moiety attached to the glyceride backbone replacing the *sn*-2 positioned FA, or less commonly, to the *sn*-1 position (Figure 1) [7]. Attaching the drug moiety to the phosphate group was used with the aim to avoid PL-prodrug hydrolysis by the enzyme PLA_2_ [33,34], and hence allowing the PL-drug conjugate to be absorbed and incorporated into the metabolic PL processing pathways. Conjugation strategy in this case usually involves hydroxyl group of the drug for linking to the phosphate group. In contrast, a different design, using the PL *sn*-2 position of the prodrug and taking advantage of PLA_2_-mediated prodrug activation became increasingly employed [28,29,30,35,36,37,38]. When linking the drug to the *sn*-2 position of the PL several synthetic techniques can be applied; a common method involves protecting the amino group of the linker, and connecting it to the *sn*-2 position of lysophospholipid, followed by removal of the protection and conjugation to the drug moiety. An optimal linker may result in a PLA_2_-sensitive prodrug [30,35]. In the following section, we will provide insights into promising therapeutic applications of the different PL-drug conjugates.

**Figure 2:**
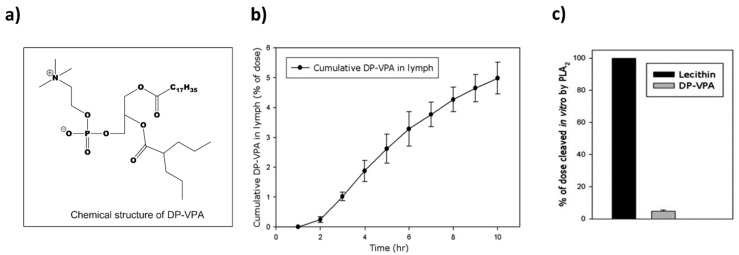
(**a**) structure of valproic acid prodrug (DP-VPA); (**b**) % of DP-VPA dose recovered in the rat lymph following oral DP-VPA dose (100 mg/kg) in LCT formulation; (**c**) PLA_2_-mediated activation of DP-VPA vs. phosphatidylcholine *in-vitro*. Reproduced with permission from reference [28], Elsevier, 2008.

Conjugation of the active drug moiety to the *sn*-2 position of the PL results in PL-based conjugate that has similar surface properties and aggregation performance like natural PL [39]. Such an approach was taken with valproic acid that was directly conjugated to the *sn*-2 position of the PL (Figure 2a) [28]. The prodrug of valproic acid and PL (named DP-VPA) was designed to pass the BBB as an intact prodrug, and since brain PLA_2_ is increased during epileptic seizure activity, to allow the release of free valproic acid at the site of epileptic focus by the action of PLA_2_ [28,40,41]. Targeting the drug to the exact site of seizure could decrease the drug dose required to accomplish pharmacological effect, and by doing so improve the systemic toxicity of VPA. As mentioned earlier, direct conjugation sometimes leads to lymphatic drug transport that bypasses portal blood flow. In the case of DP-VPA 10–18% of the postprandial orally administered dose in dogs was lymphatically transported [25]. Consequent study in rats revealed that up to 5% of the DP-VPA dose was transported via the lymphatics (Figure 2b). Taking into account that the systemic bioavailability was 8.8%, the portion of absorbed DP-VPA associated with lymphatic transport was determined to be 60%. In addition, since the presence of long chain triglyceride (LCT) in the GI lumen encourages chylomicron synthesis and overall lymphatic transport [24,42,43], the absorption of DP-VPA from LCT vs. medium chain triglyceride (MCT) vehicles, as well as food effect, was studied. DP-VPA demonstrated 3-fold increased oral absorption from LCT formulation compared to MCT, validating the importance of chylomicrons and lymphatic transport in the absorption of DP-VPA. Postprandial state resulted in 3-fold increased absorption as well, which further confirms the lymphatic transport route. This significant drug transport via the intestinal lymphatic system reveals the importance of the structural similarity between the PL-prodrug and natural PL [28].

This approach included direct conjugation between the valproic acid and the PL, without linker in the middle of the two moieties, which resulted in very low hydrolysis rate by the PLA_2_ (Figure 2c); this feature greatly influences the fate and traffic of the PL prodrugs in the body. This was also evident *in-vivo* by similar PK profile of DP-VPA in PLA_2_ knockout mice (C57BL/6) vs. control animals (BALB/c), indicative of the prodrug resistance to PLA_2_ hydrolysis [28]. It was demonstrated that the prodrug enters the enterocyte as the intact complex, associates with chylomicrons within the enterocyte, and undergoes lymphatic transport [28].

**Figure 3:**
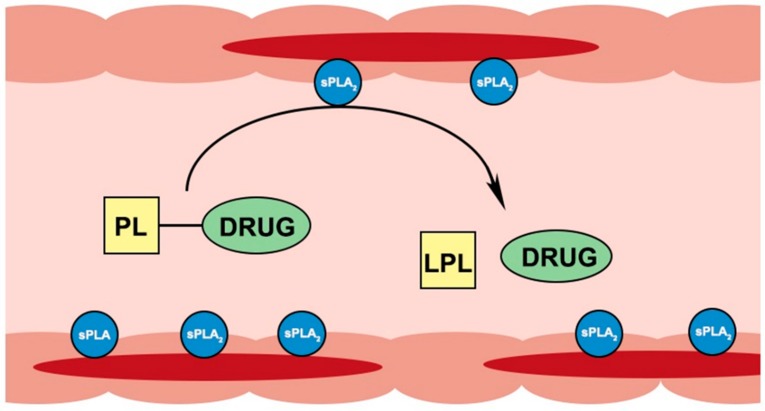
Illustration of PL-drug conjugates activation in the sPLA_2_ overexpressing inflamed intestinal tissues of IBD patients.

Secretory PLA_2_ enzyme was shown to be considerably overexpressed in areas of inflamed intestinal tissues of IBD patients [15,16,44,45]. Our group proposed to exploit this PLA_2_ overexpression for drug targeting by PL-based prodrug; orally administered conjugates can travel along the intestinal lumen until they reach the PLA_2_-rich inflamed tissue(s) where they will be activated by PLA_2_ and will release a free drug at the specific site(s) of action (Figure 3). Besides IBD, this approach can be utilized for different conditions with overexpression of PLA_2_, e.g., atherosclerosis [46], rheumatoid arthritis [47] and colon cancer [48].

**Figure 4:**
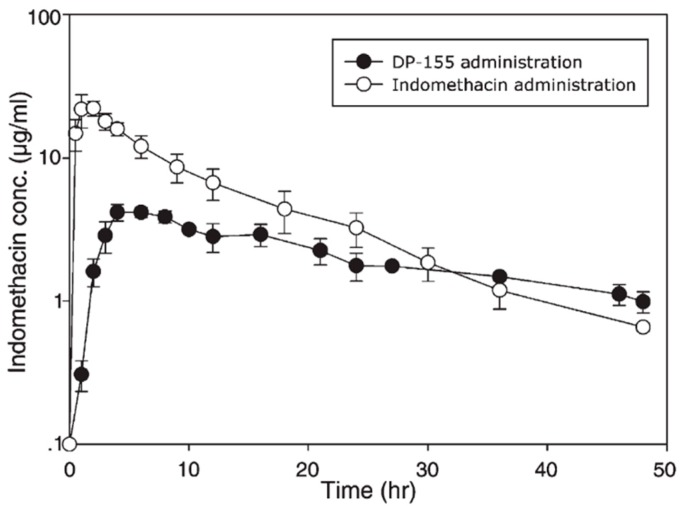
Indomethacin plasma profiles following oral administration of PL-prodrug DP-155 (●) vs. free indomethacin (○). Reproduced with permission from reference [29], Elsevier, 2007.

Absence of PL prodrug activation by the PLA_2_ is attributable to steric hindrance between the drug moiety and the PL [39]. Using a particular linker that would increase the distance between the drug moiety and the PL could resolve this lack of activation. Previously, this approach was demonstrated for PL-indomethacin [29,49] and PL-diclofenac [30,50] conjugates with several different linker lengths between the *sn*-2 carbon atom of the PL and the drug. In contrast to valproic acid, following oral administration to rats of the PL-indomethacin with 5-carbon linker (DP-155), free drug was released from the prodrug by the action of PLA_2_ within the intestinal lumen (with no absorption of intact DP-155); indomethacin terminal slope following oral administration of prodrug vs. the free drug (Figure 4) demonstrated that liberation of free indomethacin from the prodrug complex, and not the elimination half-life from the body, is the rate-controlling step [29]. This flip-flop kinetics in which the drug release from the prodrug is slower than the rate of elimination results in a controlled release (CR) drug profile in the systemic circulation; after oral administration of DP-155, the AUC of free indomethacin decreased 2-fold, *C*_max_ 5-fold and *T*_max_ was delayed 2-fold, resulting in less fluctuations in the drug plasma concentrations, which can be advantageous in cases of concentration-dependent side effects [29]. In contrast, following the administration of a prodrug with shorter linker (2-carbon length; DP-157), the liberation of free indomethacin was 20-fold lower [29].

**Figure 5:**
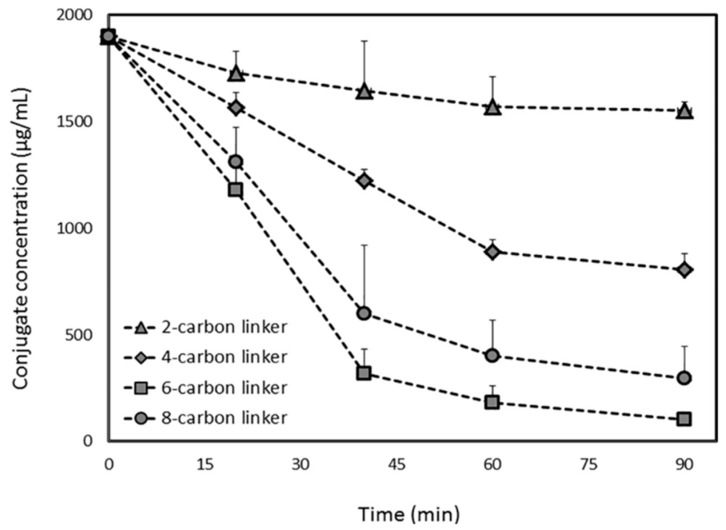
PLA_2_-mediated *in-vitro* activation of PL-diclofenac conjugates with linker lengths of 2, 4, 6 and 8-carbons. Reproduced with permission from reference [30], Elsevier, 2017.

The PL-diclofenac conjugates demonstrated similar trend: 2- and 4-carbon linker PL-diclofenac conjugates were hydrolyzed to a lower extent than the 6-carbon conjugate, whereas longer linker reduced the affinity towards PLA_2_ (Figure 5). The 6-carbon linker released ~95% of the free drug after *in-vitro* incubation with bee venom PLA_2_, whereas the 2-carbon linker prodrug released only ~20% of the free diclofenac post-incubation, showing that the 6-carbon linker is the optimal linker length in case of diclofenac PL-based prodrugs (Figure 5) [30]. These studies demonstrated that the activation of the PL-indomethacin and PL-diclofenac prodrugs by PLA_2_ enzyme is highly dependent upon linker length.

Recently, PL conjugates of antiviral oligopeptides in the *sn*-2 position of the PL, were studied [38]. These PL-prodrugs were entirely hydrolyzed by bee venom PLA_2_, releasing the free oligopeptides in the same rate of reaction as physiological PL. It was shown that the α-methylene group (a part of the oligopeptides structure) attached to the PL *sn*-2 position, has a crucial role in PLA_2_ activation, as peptidophospholipids that do not contain this functional group failed to be activated by the enzyme. When it comes to PL-prodrugs, this α-methylene group adjacent to the *sn-*2-ester functional group of the substrate can be viewed as a spacer/linker that enables conjugate-enzyme binding, required to achieve PLA_2_-mediated hydrolysis and oligopeptide liberation. The same group synthesized *sn*-2 substituted PL-prodrugs with oligo(ethylene glycol) derivatives with chain-terminal groups of NSAID drugs (sulindac, diclofenac and indomethacin) [51]. Bee venom PLA_2_ hydrolysis resulted in a series of oligo(ethylene glycol) conjugates of the NSAID drug. Once again, it was shown that a particular spacer between the PL and the drug moieties is needed for PLA_2_-mediated activation of the prodrug.

**Figure 6:**
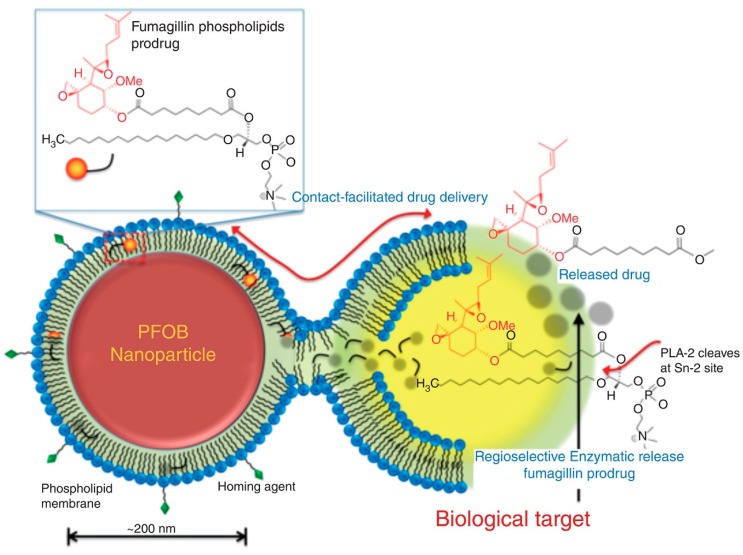
Illustration of contact-facilitated drug delivery mechanism where lipid membrane of PL-fumagillin containing nanoparticles. Binding of the nanoparticle to the targeted cell creates hemifusion complex between the two membranes, and PL-fumagilin prodrug is released into the cell, where local PLA_2_ liberates the free drug into the cytosol. Reproduced with permission from reference [36], John Wiley and Sons, 2015.

The prodrug of PL and anti-angiogenesis mycotoxin, fumagilin was designed and synthesized in order to overcome fumagilins’ poor photo-stability, poor retention in the blood and to achieve efficient intracellular transport via contact-facilitated drug delivery [32,36,52]. Fumagilin contains highly reactive epoxide groups, responsible for its pharmacological activity, which were conserved by saponifying fumagilin to fumagiol in the process of prodrug synthesis. Chemical instability is the reason for poor retention of fumagilin in the circulation and it is due to the photosensitive decatetraenedioic tail; this was resolved by substituting the tail with the 7-carbon acyl linker at the *sn*-2 position of the PL. Native fumagillin was delivered using a nanocarrier system targeted to angiogenic endothelium expressing α_v_β_3_-integrin. Fusion of the fumagilin prodrug inside a α_v_β_3_-targeted perfluorocarbon (PFC) nanocarrier system enabled the endocytosis and drug delivery followed by passive transfer to the targeted cell [32]. Once inside the cell, the intracellular PLA_2_ is responsible for prodrug activation and release of the free drug from the *sn*-2 position (Figure 6). *In-vivo* studies demonstrated decreased angiogenesis with nanoparticles containing fumagilin prodrug compared to the control nanoparticles [32]. In addition, the contact-facilitated drug delivery approach has been utilized in development of enhanced treatment of multiple myeloma [53]. A novel PL conjugate of small molecule inhibitor of MYC-MAX dimerization (MYC-b-HLHZIP transcription factor c-Myc; MAX- bHLHZIP protein) was synthesized. Developed inhibitors are small molecules that have difficulties inhibiting protein-protein/protein-DNA interactions. The prodrug itself had upgraded potency towards myeloma cell cultures comparing to the free drug. After incorporating such molecules in integrin-targeted nanoparticles, the prodrug effect was tested on mice model of myeloid leukemia. Nanoparticles carrying the prodrug showed substantially longer animal survival when compared to controlled nanoparticles (both targeted and untargeted) [53]. When administered systemically as free compounds neither the Myc-inhibitor 1, nor the PL-prodrug exhibited any effect. Even more recently, prodrugs of docetaxel and fumagilin incorporated in α_v_β_3_-micellar nanotherapy demonstrated direct anti-angiogenesis effect and can ameliorate asthma in the rat model. Most common symptoms of asthma airway hyper-responsiveness and increased microvascularity, were considerably reduced with both treatments [54]. The combination of PL-prodrugs and targeted formulation approaches in these cases is crucial. An interesting utilization of PL in a formulation approach was recently demonstrated using cell membrane-coated nanoparticles for achieving targeting, long blood circulation, and immune escaping [55,56,57]. Cell membrane-coated nanoparticles are a biomimetic tool consisting of a nanoparticle core coated with membrane derived from natural cells, i.e., erythrocytes [58,59,60], platelets [61], white blood cells [62], cancer cells [63], stem cells [64], or microbial cells [65]. Recent study describes the combination of prodrug approach (dimeric paclitaxel prodrug) and nanoparticles coated with red blood cell membrane [66]. Although the formulation approach is beyond the scope of this article, this research field is worth mentioning since it can be used together with the PL prodrug approach, allowing higher success than one approach by itself.

As mentioned above, drugs can also be linked to PL through the phosphate group. Majority of the drugs conjugated this way are nucleosides with antiviral or antineoplastic pharmacological effect, which are characterized by poor absorption and suboptimal pharmacokinetic behavior. Resistance towards nucleoside analogs is very common, attributable to either decreased expression of transport proteins, which leads to lower uptake of drugs (i.e., human equilibrative nucleoside transporter 1 (hENT1), loss of deoxycytidine kinase (dCK) expression and consequent decrease in drug activation, P-glycoprotein (P-gp) increased efflux activity, and cytidine deaminase loss of function (cytarabine, gemcitabine) or nuclear exonucleases/ribonucleotide reductase (fludarabine) [67,68]. Conjugation with PL can improve passive permeability of such hydrophilic drugs, and bypass active ligand transport mechanisms and transport resistance barriers [5,68]. One way to overcome drug resistance is reducing the drug efflux via transporters e.g., the multi drug resistance (MDR) transporters, resulting in increased accumulation within the cell [33]. In some cases PL-nucleoside conjugates can overcome possible deficiencies in nucleoside kinase activity by liberating the drug moiety in a monophosphate form inside the cell, to avoid dependency on dCK activation [67,68].

PL-gemcitabine prodrugs, with the drug conjugated through the phosphate group were investigated; the aim of the design of these conjugates was to bypass multiple resistance pathways, inhibit protein kinase C (PKC), improve pharmacokinetic profile and increase brain penetration. Kucera et al. designed PL prodrug with gemcitabine conjugated through the phosphate group with thioether functional group in the *sn*-1 position and oxyether group in the *sn*-2 position of the PL [33,69]. PL-gemcitabine prodrugs, in contrast to the free drug, did not enter the cell via hENT1 transporter, was not substrate for MDR-1 efflux pump, and dCK was not needed for gemcitabine prodrug activation, thereby bypassing three resistance mechanisms of gemcitabine, that are largely responsible for its ineffectiveness in cancer therapy [33]. Consequently, a similar prodrug of gemcitabine was synthesized with an amido functional group in the *sn*-1 position of the phospholipid (KPC34), which resulted in more potent cytotoxicity [70]. The advantage of KPC34 in comparison to gemcitabine is that it can be given orally, whereas gemcitabine is only effective once given intraperitonealy. In a recent study involving preclinical models of Philadelphia chromosome positive B cell acute lymphoblastic leukemia, KPC34 was shown to be orally bioavailable, overcome multiple resistance mechanisms, inhibit the various PKC isoforms and cross the BBB [71]. KPC34 administration following doxorubicin and cytarabine treatment in animal models achieved reversed hind limb paresis (multiple times), showing the KPC34 effect in CNS leukemia [71]. It has been suggested that KPC34 enters lipoprotein complexes, which may pass the BBB in CNS involved leukemia [72,73,74]. Promising results have been shown in animal model of acute myeloid leukemia (AML) as well as improved CNS leukemic burden [75].

The use of the *sn*-1 bond of PL was demonstrated for anticancer ether lipids (ProAELs) where drug was linked to the *sn*-1 position of PL through an ether bond [76]. Such lipids can be formulated as liposomes, which are subjected to sPLA_2_-mediated activation, where proAELs release anticancer ether lipids (AELs) thereby producing a free hydroxyl group in the *sn*-2 position [77]. Since such lipids do not support structure of liposomes, the liposomes collapse and the ether lipids are released specifically in the site of cancer tissue. This approach leads to increased chemical stability, and demonstrates cleavage with PLA_2_, but somewhat modest efficacy in tumor growth reduction [31]. Additionally, proAELs with a thio-ester bond in the *sn*-2 position of the PL were synthesized. PLA_2_-mediated activation was shown to be slower, and hence the cytotoxicity was lower as well, than the above mentioned proAELs [78]. The rationale for this decreased activation by the enzyme is explained by molecular dynamics simulations, presented hereinafter (Section 4). Clinical studies of PL-ether analog for multiple myeloma and other hematologic malignancies are currently conducted [79].

Altogether, the key advantages of designing PL-based prodrugs are based on their ability to incorporate certain PL processing pathways, resulting in lymphatic transport, controlled release profile of tested drugs, brain permeation, overcoming resistance mechanisms by bypassing transport proteins, and providing an opportunity for exploiting PLA_2_ that is overexpressed in certain diseased tissues, thereby providing an opportunity for site-specific drug release.

## 4. Computational Modeling of PL-Based Prodrugs

Nowadays, novel molecular modeling technologies are available at aiming to evaluate PLA_2_-mediated activity towards different PL-drug conjugates, as well as to predict which structural conjugate modifications are a prerequisite for the prodrug to undergo enzymatic hydrolysis, thereby reducing the time needed for experimentation [37,50]. Molecular dynamics (MD) simulation was previously used to explain why PLA_2_ specifically catalyzes the hydrolysis of the fatty acid ester bond at position *sn*-2, but not at *sn*-1 position, of the PL and as a valuable mean for assessing sPLA_2_ activity toward different PL-drug substrates [80,81]. MD simulations technique is well established for elucidation of macromolecular structure-to-function relationships [82]. Explaining the dynamic macromolecular features enables successful analysis of conformational ensembles. MD simulations are proven to be useful and biologically relevant.

**Figure 7:**
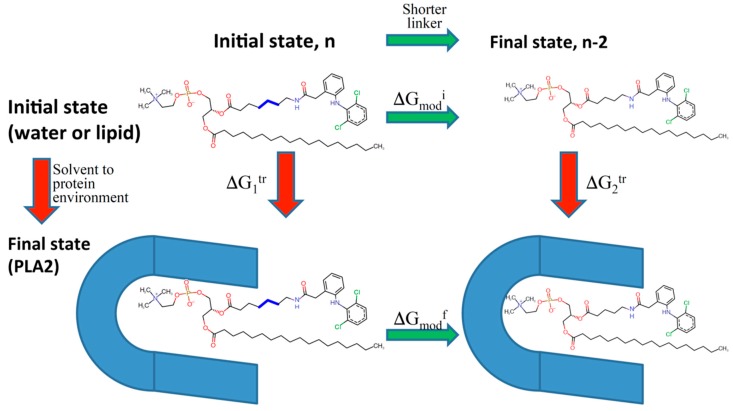
Thermodynamic cycle diagram used in the computational modeling of relative binding free energy of PL-diclofenac conjugates to the PLA_2_ enzyme, using the difference between the final state (conjugate in PLA_2_, Δ*G_mod_^f^*) and the initial state free energy (conjugate in water/lipid, Δ*G_mod_^i^*)*.* Δ*G_1_^tr^* and Δ*G_2_^tr^* represent free energies of adding/removing –CH_2_ units from the linker. Reproduced with permission from reference [50], Springer Nature, 2017.

Using the modern computational methodology based on Thermodynamic integration and Weighted Histogram Analysis Method (WHAM)/Umbrella Sampling method, our group designed several PL-diclofenac and PL-indomethacin conjugates with different linker lengths between the *sn-*2 positioned carbon in the PL and the drug moiety [49,50]. This *in-silico* approach was established to predict the alterations in the PLA_2_ transition state binding free energy of the prodrugs while the linker length is increased/decreased (Figure 7). We gained an excellent correlation between computational simulations and experimentally obtained results for both PL-indomethacin and PL-diclofenac prodrugs. For PL-indomethacin, it was confirmed that the prodrug with linker length 5-carbon atoms has the greatest extent of activation by PLA_2_, and hence is considered optimal for this prodrug. Similarly, for PL-diclofenac prodrugs the optimal linker length, with highest rate of activation was 6-carbon linker, whereas linker with 2-carbon atoms had lowest degree of activation.

It should be noted that optimal length of the linker can be determined only on a case-by-case basis, since the drug structure affects the steric interactions with the enzyme. Altogether, this further demonstrates that the length of the linker plays a crucial role in the successful activation by PLA_2_, and determines the amount of the free drug that will be released from the complex.

A similar approach was used for retinoic acid (ATRA) [37]. Direct conjugation between ATRA and *sn*-2 position of the PL resulted in the lack of PLA_2_-mediated activity. This is explained by the rigid structure of ATRA, which contains methyl group next to the carboxyl functional group, which is in contrast to physiologically-occurring FAs (flexible molecules, usually without branching). Consequently, a 6-carbonic linker was incorporated between the PL and ATRA, and hydrolysis was demonstrated by MD simulations. It was demonstrated that the prerequisite for sPLA_2_-mediated hydrolysis is stable Michaelis-Menten complex and a water molecule, that acts as a nucleophile and is free to enter the catalytic site [37]. Introduction of 6-carbon linker between ATRA and the PL resulted in stability of Michaelis-Menten complex and presence of water molecule, as opposed to the prodrug with direct conjugation. This computational prediction was further confirmed experimentally, and incorporation of 6-carbon linker enabled retinoids to overcome the lack of PLA_2_ activity, that originated from the rigid nature of ATRA [83,84].

To gain further insight into the mechanism behind the lower PLA_2_ activity *in-vitro* towards proAELs with a thioester bond in the *sn*-2 position, MD simulations and density functional theory calculations were conducted [78]. The MD simulations demonstrated perfect fit of both natural substrate with ester bond and proAELs with a thioester within the active site of PLA_2_; water molecule was able to reach the active site freely for both substrates. However, density functional theory described the variance in the hydrolysis rate between the two substrates as an inherit electronic difference among sulfur and oxygen atom [78].

Overall, computational modeling allows the structural optimization of the PL-prodrugs, providing the development process that is less empirical and more efficient. Correlation between *in-vitro* and *in-silico* studies encourages the utilization of molecular modeling techniques for activity predictions of newly designed compounds prior to conducting experiments.

## 5. Discussion

**Figure 8:**
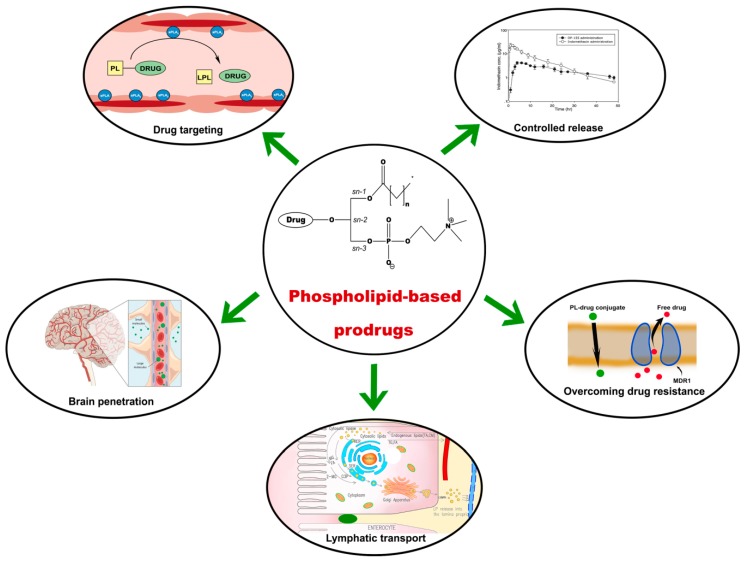
Illustration of the major challenges and potential solutions afforded by the PL-based prodrug approach.

Phospholipid prodrugs present a diverse and promising class of carriers, whose future utilizations holds a promise for various optimization and developing approaches (Figure 8). One of the main advantages of creating a PL prodrug is the capability to taking advantage of endogenous metabolic pathways of PL, hence a good understanding of physiological pathways is a necessary prerequisite for efficient prodrug design.

In contrast to triglyceride and certain formulation approaches, directing the PL-drug conjugates to lymphatic transport is rare, however we believe this is still underexplored field with great prospective. PL-prodrug can undergo lymphatic transport thereby bypassing extensive first-pass hepatic metabolism. This allows prodrugs to avoid presystemic metabolism and reach higher systemic bioavailability. Lymphatically transported prodrugs could be crucial for drug targeting exclusively to diseased lymphatic tissue, or preventing/treating tumor metastases dissemination. PL-prodrugs can allow non-absorbable drugs to be orally bioavailable, as well as allowing manipulation of delivery rate to the blood, e.g., controlled drug delivery. However, it should be noted that in specific disease the uptake of lipoproteins from the lymphatics can vary, as well as PL-drug conjugate partitioning from lipoproteins to target cells [20].

An important advantage that was achieved by PL-based prodrugs is overcoming cancer resistant to chemotherapeutic agents. PL-nucleoside analogs were able to overcome chemoresistance, turning the drug penetration into the cells a passive process (diffusion), as opposed to active drug uptake, where carrier-mediated uptake is one of the main reasons of resistance. Additional advantages of these prodrugs include longer blood circulation and increased stability. However further research is needed for understanding the pharmacokinetics and intracellular activation. In the future, methods for increasing cytotoxicity and reducing side effects should be investigated, e.g., by conjugation with antibodies, or creating multi-drug complexes. Rational drug/prodrug combination, and improved understanding of pharmacokinetics, mechanism of action and safety profile is a key for improved clinical outcomes.

As presented above, PL-based prodrugs can be designed to take advantage of PLA_2_, and since this enzyme is not selective toward specific FA, successful PLA_2_-mediated activation may be achieved with careful design. The PL-indomethacin study clearly shows controlled release of the free drug. This ability of the PL prodrugs could be clinically relevant for patient compliance, since it can allow to less frequent dosing of the drug. In addition, such prodrugs can be formulated even as a liquid dosage form, and still provide a controlled-release drug profile. This could be especially advantageous for pediatric/elderly populations in which swallowing of a solid dosage form is impossible or painful.

PL-drug design may include various conjugation strategies and employment of linkers between the PL and the drug, which may significantly affect the prodrug properties, together with the release of the free drug from the complex, GI stability, and targeting specific enzymes. It is important to understand the significance of the spacer between the drug and the PL moieties in order to accomplish effective activation by PLA_2_. Combination of optimal prodrug design and a smart choice of delivery system may provide successful drug targeting and sufficient quantity of drugs at the site of action.

Optimal prodrug design and development involve numerous steps and variables, and as such, empirical approach can be time and effort-consuming to significant degree. Modern computational approaches can be used for more intelligent design of advanced prodrugs. The novel molecular modeling approach presented in this work has been shown to have the potential to predict the optimal structural design of prodrugs intended to be activated by PLA_2_. Modern computational modeling can allow prediction of the optimal prodrug design and may help to achieve the desired properties, meanwhile significantly decreasing the number of syntheses and/or experiments needed.

In summary, different approaches in the design of PL-prodrugs may be taken, aiming to achieve specific goals. As illustrated in Figure 8, a number of applications can be pursued: targeting inflamed/cancerous tissues, lymphatic transport, passing the BBB, overcoming resistance to nucleoside analogs, or modify PK profile. When lymphatic transport/targeting is aimed for, resistance to PLA_2_-mediated hydrolysis is desired. This can be achieved by direct linking of the drug moiety to the glyceride backbone, with no (or very short) linker, that prevent prodrug hydrolysis due to steric hindrance. When cancer resistance is relevant, conjugating the drug to the phosphate group was shown beneficial for PL prodrugs of nucleoside analogs; however slight alteration in the PL moiety demonstrated different anticancer effect (for instance, amido group showed higher potency then tio ether group in the *sn*-1 position of the PL) [70]. The effect of structural changes was also shown in a recent study of the isoprenoid acids prodrugs for anticancer activity, where the drug was incorporated to *sn*-1 and *sn*-2 position of the PL; short chain isoprenoids were more potent when occupy only one position, whereas longer ones were active when both *sn*-1 and *sn*-2 positions are occupied [85]. In addition to this, the number of unsaturated bonds in the isoprenoid moiety also has an effect on the prodrug activity. Conjugating the drug to the phosphate group may allow enhanced BBB penetration [71]. In cases when PLA_2_-mediated activation is desired for targeting cancer or sites of inflammation, a specific linker/spacer should be used, in order to yield optimal release profile. Numerous linker types can be used from carbonic linkers, peptides, PEGs, or other synthetic spacers, adjusted for specific therapeutic need. A good example of this is that ProAELs synthesized with thio-ester bond in the linker achieve lower rate of hydrolysis when compared to natural substrate of PLA_2_ [78]. A ligand conjugated to PL through a PEG linker was used to develop long-lasting ligands that could bind to the proteins of the cell membrane, by constraining the subcellular localization of the ligand [86].

Altogether, a careful design of PL-prodrugs targeted for particular disease/condition gives rise to novel therapeutic tools that can improve overall patient care.

## 6. Conclusions

This work presents an overview of PL-based prodrugs, their recent applications, prospects and challenges, and molecular modeling approaches for optimization of the prodrug design. Conjugation with PLs allows the prodrug to incorporate into certain PL processing pathways, undergo lymphatic transport, pass BBB, deliver controlled release blood profile, overcome resistance mechanisms by bypassing transport proteins, and may exploit PLA_2_-mediated activation, resulting in site-specific drug release. The research and use of this promising approach is expected to grow in the coming years.
